# Supplementary material for: Effects of supplemental irrigation at the jointing stage on population dynamics, grain yield, and water-use efficiency of two different spike-type wheat cultivars
Source: PLoS One. 2020 Apr 7;15(4):e0230484. doi: 10.1371/journal.pone.0230484 (PMC7138302; doi:10.1371/journal.pone.0230484)
Supplement: Attachment — Submitted filename: Response to Reviewers.docx [file pone.0230484.s001.docx]

**Cover letter to editor**

Dear Prof. Zhang,

Thank you for your kind consideration,

We have carefully revised and corrected the Manuscript ID PONE-D-19-34837 entitled “Effects of supplemental irrigation on population dynamics, grain yield, and water use efficiency of two different spike-type wheat cultivars at jointing stage” which we submitted to *PLOS ONE,* according to your comments and those of the reviewers.

**Funding Statement**

The commercial company of Zibo HeFeng Seed Technology co., ltd. just provided support in the form of salaries for authors [Dong Wang], but did not have any additional role in the study design, data collection and analysis, decision to publish, or preparation of the manuscript. So we deleted the commercial company in the new revision. The **Funding Statement** in the new revised manuscript was described as follows:

This work was financially supported by the Major Scientific and Technological Innovation Project of Shandong Province (2019JZZY010716), the Taishan Industry Leader Talent Project of Shandong, and the Special Fund for Agro-scientific Research in the Public Interest (201503130).

**Competing Interests Statement**

There was no competing interests exists in this paper.

Responses to the reviewers' comments are presented point by point below.

**Responses to Reviewer #1**

Following the comments’ order:

The new revision had been edited by the American Journal Experts (AJE) which was recommended by the PLOS editorial team. The changes indicated by red font, can be seen in the new version directly.

According to the Reviewer’s suggest, we had replaced the figures by the higher-resolution ones in the new revision (Fig. 1–5 in the new revision).

**Discussion**

We had rephrased the discussion and also added one paragraph at the end of the Discussion to clarify the differences between large- and multispike cultivars under different wetting layer treatments (Line 269–324 in the new revision).

**Responses to Reviewer #2**

Following the comments’ order:

**Question:** The apical leaf Pn of main stem, I, and IV tillers were measured, how about other tillers?

**Answer:** The spike number per plant is 1.6–2.5 stem in the large-spike cultivar SN23, and 2.9–3.5 stem in the multispike cultivar SN29. In order to reduce the workload, only representative tillers were selected for the measurement. Therefore, we only measured the main stem, tillers I and IV, where the tiller I represent as the productive tiller and the tiller IV represent as the inefficient tiller.

The new revision had been edited by the American Journal Experts (AJE) which was recommended by the PLOS editorial team. The changes indicated by red font, can be seen in the new version directly.

**Abstract**

Line 49: The last sentence in the Abstract had been rewritten as “Irrigation at the jointing stage brought the soil water content in the 0–20 cm profile to 100% of field capacity, making it the most suitable supplemental irrigation regime for both the large- and multispike cultivars in the North China Plain” (Line 30 in the new revision).

**Discussion**

According to the Reviewer’s suggest, one passage was added at the end of the Discussion to clarify the differences between large- and multispike cultivars under different wetting layer treatments (Line 307–324 in the new revision).

**Tables**

Table 4: According to the Reviewer’s suggest, we deleted it and updated the order of Tables.

Table 6: The significance according to the LSD test performed had been labeled (Table 5 in the new revision).

**References**

Line 346: “272–273 (2019) 12–19” was changed to “2019; 272–273: 12–19” (Line 349 in the new revision).

Line 347: “Ha, GJ” was changed to “Han GJ” (Line 350 in the new revision).

Line 368: “Plant Population Effects on Growth and Yield in Water-Seeded Rice” was changed to “Plant population effects on growth and yield in water-seeded rice” (Line 371 in the new revision).

**Based on our checking carefully, other revisions also were made:**

The changes indicated by red font, can be seen in the new version directly.

**Title**

Line 1–2: the title was rephrased by “Effects of supplemental irrigation at the jointing stage on population dynamics, grain yield, and water-use efficiency of two different spike-type wheat cultivars” (Line 1–2 in the new revision).

**Introduction**

Line 70–94: The part of Introduction had been rephrased in the new revision (Line 51–71 in the new revision).

**Measurements**

**Soil water content and water consumption by winter wheat**

Line 171–176: The calculation of the total water consumption of winter wheat has been rewritten as “The total water consumption of winter wheat was calculated using the soil water balance equation [27]: ET = ΔS + I + P – R – D + CR, where ET (mm) is the total water consumption, ΔS (mm) is the water consumption from the 0–200 cm soil layer during the growing season, I (mm) and P (mm) are the irrigation and precipitation, respectively, R (mm) is the runoff, D (mm) is the drainage from the root zone, and CR (mm) is the capillary rise to the root zone. Three factors in this equation (R, D and CR) can be ignored in this experimental site” (Line 142–147 in the new revision).

**Population dynamics of winter wheat**

Line 178–183: The marking and sampling method of tillers and the method of population survey have been rewritten as “Tillers were marked from the appearance of the first tiller in wheat. The newly initiated tillers of each plant were checked and tagged every 5 days. After jointing, the tagged plants were selected and separated according to the tiller positions for measuring. In this paper, the main stem is represented by O, and the primary tillers growing from the true leaf axillary of O are represented by I, II, III, IV, etc. Conversely, I-p, I-1, I-2, etc. are used for the secondary tillers growing from the axillary of the true leaf of the primary tillers [28]. The number of tillers (stems) per square meter was investigated at wintering, re-greening, standing, jointing, anthesis and maturity. At jointing, the number of tillers (stems) was investigated from 0 days after jointing to anthesis and it was checked once every 7 days” (Line 149–157 in the new revision).

**References**

Eight references were deleted and six new references were added in Line 389–398, Line 409–410, and Line 419–423 in the new revision, respectively.
